# Author Correction: Far-UVC light: A new tool to control the spread of airborne-mediated microbial diseases

**DOI:** 10.1038/s41598-021-97682-w

**Published:** 2021-09-07

**Authors:** David Welch, Manuela Buonanno, Veljko Grilj, Igor Shuryak, Connor Crickmore, Alan W. Bigelow, Gerhard Randers-Pehrson, Gary W. Johnson, David J. Brenner

**Affiliations:** Center for Radiological Research, Columbia University Medical Center, New York, New York 10032 USA

Correction to: *Scientific Reports* https://doi.org/10.1038/s41598-018-21058-w, published online 09 February 2018

The Authors wish to clarify the Competing Interests section, which now reads as follows:

“The authors G.R.-P, D.J.B and A.B. have a granted patent entitled ‘Apparatus, method and system for selectively affecting and/or killing a virus’ (US10780189B2), that relates to the use of filtered 222 nm UV light to inactivate viruses. In addition, D.J.B has an ongoing non-financial collaboration with Eden Park Illumination, and the authors’ institution, Columbia University, has licensed aspects of UV light technology to USHIO Inc.”

